# Men’s Satisfaction with General Health Services is Associated with Future Use of HIV Testing in Malawi: A Community-Representative Survey

**DOI:** 10.1007/s10461-024-04352-9

**Published:** 2024-06-13

**Authors:** Marguerite Thorp, Kelvin Balakasi, Shaukat Khan, Christian Stillson, Joep J. van Oosterhout, Brooke E. Nichols, Morna Cornell, Kathryn Dovel

**Affiliations:** 1 David Geffen School of Medicine, University of California Los Angeles, Los Angeles, USA; 2 Partners in Hope, Lilongwe, Malawi; 3 FIND, The Global Alliance for Diagnostics, Geneva, Switzerland; 4 Clinton Health Access Initiative, Washington, DC USA; 5 Centre for Infectious Disease Epidemiology and Research, University of Cape Town, Cape Town, South Africa

**Keywords:** HIV testing uptake, Sub-Saharan Africa, Healthcare quality, Patient satisfaction, Multivariable regression

## Abstract

Across sub-Saharan Africa, men are less likely to know their HIV status than women, leading to later treatment initiation. Little is known about how experiences with general health services affect men’s use of HIV testing. We used data from a 2019 community-representative survey of men in Malawi to understand frequency and cause of men’s negative health service experiences (defined as men reporting they “would not recommend” a facility) and their association with future HIV testing. We conducted univariable and multivariable logistic regressions to determine which aspects of health facility visits were associated with would-not-recommend experiences and to determine if would-not-recommend experiences 12–24 months prior to the survey were associated with HIV testing in the 12 months prior to the survey. Among 1,098 men eligible for HIV testing in the 12 months prior to the survey, median age was 34 years; 9% of men reported at least one would-not-recommend experience, which did not differ by sociodemographics, gender norm beliefs, or HIV stigma beliefs. The factors most strongly associated with would-not-recommend experiences were cost (aOR 5.8, 95%CI 2.9–11.4), cleanliness (aOR 4.2, 95%CI 1.8–9.9), medicine availability (aOR 3.3, 95%CI 1.7–6.4), and wait times (aOR 2.7, 95%CI 1.5-5.0). Reporting a would-not-recommend experience 12–24 months ago was associated with a 59% decrease in likelihood of testing for HIV in the last 12 months (aOR 0.41; 95% CI:0.17–0.96). Dissatisfaction with general health services was strongly associated with reduced HIV testing. Coverage of high-priority screening services like HIV testing may benefit from improving overall health system quality.

**Supplementary Information:**

The online version contains supplementary material available at 10.1007/s10461-024-04352-9.

## Introduction

Globally, men have higher rates of morbidity and mortality than women across most disease categories [1]. These gender disparities are partly due to men’s lower access to and use of health services, including screening and preventative services [2, 3]. In sub-Saharan Africa, men are less likely than women to know their HIV status [4], which contributes to lower rates of antiretroviral therapy (ART) initiation and higher rates of HIV-related morbidity and mortality [5–7]. Gender disparities in HIV services directly affect the health of men and increase risk of HIV acquisition among women [8]. To increase men’s use of HIV services and improve health outcomes, it is critical to understand what influences men’s uptake of screening services such as HIV testing.

Individuals in all settings make complex decisions about seeking health services. Care recipients’ previous experiences with health services are key to these decisions [9, 10]. Throughout sub-Saharan Africa, clients report negative experiences with health services, including rude or punitive behavior from health care workers (HCWs), long wait times, limited information, and lack of holistic care [11, 12]. Poor quality services are associated with low patient satisfaction [13], and preliminary evidence suggests that low satisfaction is associated with reduced use of services [14]. However, the literature on client satisfaction in the region has mainly focused on maternal health services [15, 16]; little is known about how men’s experiences with services influence future use of care.

There has been extensive research on factors associated with men’s use of HIV testing services, especially on harmful gender norms and stigmatizing beliefs about HIV that may discourage men from care-seeking or structural and economic barriers to testing [17–20], but few studies account for the role of previous experiences with general health services. Some qualitative literature explores client satisfaction with an array of HIV services [21–23], but it is unclear if satisfaction with non-HIV services impact future use of HIV testing. This gap is important because the majority of men’s experiences with health facilities are with non-HIV services. In Malawi, for example, men attend outpatient departments (OPD) regularly (mean of 2 visits within a 12-month period), with the most common reason for attending a facility being illness or injury [24]. Screening services such as HIV testing may be especially impacted by client satisfaction with general health services. Use of screening services may be perceived as non-urgent, especially for healthy men, and therefore the benefit of service utilization may not outweigh anticipated unpleasant health facility experiences [25]. Understanding how experiences with non-HIV services affect HIV testing can inform what interventions are needed to increase men’s use of screening services.

We used data from a community-representative survey with men in Malawi to understand the impact of client satisfaction with non-HIV health services on future use of general services and HIV testing, compared to the impact of gender norms, HIV stigma, and socioeconomic factors. We also explored which client characteristics, facility visit types, and components of service quality were most associated with client satisfaction.

## Methods

### Objectives

We had two overarching objectives. First, we explored client satisfaction with general health services and factors associated with satisfaction, including client demographic characteristics, facility characteristics, reasons for facility visit (i.e., men attending as a caregiver versus a primary care recipient), and components of healthcare quality. Second, we explored whether client satisfaction with general health services accessed 12–24 months ago was associated with use of HIV testing services in the past 12 months.

#### Setting, Design

We used data from a large, community-representative survey of men in central and southern Malawi that assessed how often men attended health facilities and what services were offered and received [24]. The study used a multi-staged sampling design, purposively selecting two high-burden HIV districts (Chikwawa and Lilongwe districts) and three high-burden health facilities within each district (six facilities total). Within each facility catchment area, six villages were randomly selected (36 villages total). Household census listings were used to randomly select 45 respondents from each village, using randomized number generation (Appendix A: facility map). Eligibility criteria included: (1) aged 15–64 years; (2) current resident of the participating village; and (3) spent > 15 of the last 30 nights within the village. Individual respondents were stratified by age within each village to ensure representation across three age categories. The study, which only targeted men, screened 1,293 men; 1,187 completed the survey (59 were outside the age range, 21 spent < 15 of the last 30 nights within the village, 14 could not confirm their identity, 11 were unable to consent due to disability or intoxication, and one man refused). With such a rigorous sampling frame and minimal loss of screened participants, we feel confident in the representativeness of the surveyed participants.

### Data Collection

Male research assistants traced participants in the community with the assistance of community health workers and village chiefs. Surveys were conducted at a location convenient to participants, typically their home, the village chief’s residence, or the participant’s place of work, and responses were entered into SurveyCTO using an electronic tablet. Research assistants conducted written informed consent for all participants over 18 years of age and written assent for participants 15–17 years old. Survey domains included: (1) sociodemographic characteristics; (2) previous facility visits, reason for visit, services offered and received, and perceived quality and satisfaction with the visit; (3) beliefs about HIV stigma and gender norms; and (4) HIV testing history. Men were asked to report details from their four most recent experiences at health facilities, referencing their personal health record (health “passport”) as needed. Surveys lasted approximately 55 min and were conducted in the local language. After a one-day pilot with study staff and participants at a non-participating health facility, the survey was revised accordingly. For this secondary analysis, we excluded survey data from men who reported testing positive for HIV more than 12 months ago, because their healthcare utilization would be different from the general population.

### Variables

Our primary variable of interest was client satisfaction with health services in the past 24 months. Because long recall times are prone to recall bias and the median time since reported facility visits was 6.5 months (IQR:2.4–13.0), we used one simple, novel measure to approximate overall satisfaction with the most recent four visits in the last 24 months: “Would you recommend this facility to a friend or family member?” (binary, yes/no). If men responded “no,” we considered that visit a “would-not-recommend” visit.

We assessed the relationship between having would-not-recommend visits and three dependent variables of interest: whether men reported any visit in the last 12 months, whether men returned in the last 12 months to a facility where they had a visit 12–24 months ago, and self-reported use of HIV testing services in the last 12 months.

As independent variables, we included sociodemographic characteristics such as age, marital status (married [monogamous or polygamous] vs. single, divorced, or widowed), and ever attended secondary school (yes/no). As a proxy for economic status we included two variables: having financial savings in a bank or another formal or informal savings scheme (yes/no) and a wealth index of 22 household assets that commonly reflect wealth in rural Malawi [26]. To analyze our wealth index, we used the first dimension of a principal component analysis, which we linearly transformed to a scale of 0 to 10 with a resulting mean of 1.88 (median 1.44, IQR 0.75–2.49). We also included two health-related variables that may independently impact use of health services: HIV risk, approximated by the reported number of sexual partners in the past 12 months, and self-reported health status, defined as good/very good vs. poor/very poor health.

We included measures on gender norms and HIV stigma as additional possible confounders of the relationship between satisfaction and future services used. We measured men’s acceptance of harmful gender norms with a modified version of the Gender Equitable Men (GEM) survey (shortened from 24 to 11 questions), a tool used previously in Malawi and validated in similar settings in sub-Saharan Africa [27]. We removed GEM questions that were not associated with men’s use of health services in previous studies in Malawi [28, 29]. We asked questions on a 5-point Likert scale from “strongly agree” to “strongly disagree.” We collapsed responses into four distinct measures, each including three questions: *measure 1*: violence is permissible; *measure 2*: male sexual dominance is acceptable; *measure 3*: women’s roles should be confined to the household; and *measure 4*: men control decisions in the participant’s household (Appendix B). We measured HIV stigma as a composite of six questions using the same 5-point Likert scale. HIV stigma questions, based on the Demographic Health Survey’s AIDS Indicator Survey, asked about: (1) fear of the social consequences of an HIV diagnosis (relationship dissolution, lack of future relationships, or loss of employment); (2) willingness to impose such consequences on others (willingness to end relationships, decline caregiving for a family member, or fire a local teacher if they had HIV); and (3) unwillingness to disclose an HIV diagnosis (own diagnosis to family members and family member’s diagnosis to community). For each of the four gender norms and HIV stigma, we summed participant scores using Likert scale responses. We then created dichotomous variables for each construct to measure relative acceptance of harmful gender norms or HIV stigma compared to other study participants, separating 20% of respondents with the highest degree of gender bias or HIV-stigmatizing beliefs from the remaining 80% in each category (high vs. low bias). We found no evidence of multicollinearity between the four gender norm constructs (variance inflation factors < 2.0). We also performed sensitivity analyses using the total GEM score (not shown) and results did not differ.

Men were asked additional questions about each facility visit, including the visit location. We defined “public” as Ministry of Health-run facilities; “mission or NGO” included Christian Health Association of Malawi facilities and others run by missionary or non-profit organizations; and “private” included for-profit facilities. Participants were also asked the main recipient of the service (themselves or others) and what service was received, which was grouped into six categories: outpatient department (for illness or injury), HIV-related (HIV testing and counseling and ART clinic), maternal and child health (antenatal, delivery, postpartum, and under-5 clinic), dentist, family planning (including voluntary medical male circumcision), and other.

We also included detailed questions about satisfaction with services received during men’s most recent facility visit. Men were asked whether they experienced major, minor or no problems with the following components of each health service drawn from the Demographic and Health Surveys Program’s Service Provision Assessment exit interview questionnaire [30]: cost, cleanliness, wait time, medicine availability, provider treatment, opening days and hours, privacy of exam and discussion, and the ability to discuss their concerns. For this analysis, we created a dichotomous variable (0 for minor or no problems, 1 for major problems) because it was unlikely that minor problems would significantly change client satisfaction.

### Analysis

We conducted five analyses with two primary goals: (a) to understand would-not-recommend visits by exploring associations between would-not-recommend visits and men’s characteristics, facility visit types, and specific quality problems; and (b) to understand the consequences of would-not-recommend visits on future general and HIV-specific health seeking behavior. First, we tested for differences in sociodemographic characteristics, gender norm beliefs, and HIV stigma beliefs between men who reported having a would-not-recommend visit in the last 24 months versus men who rated all their facility visits in this period as acceptable, using t-tests and Wilcoxon rank sum tests. Second, we compared the proportions of would-not-recommend and acceptable experiences that occurred at public, mission, and private facilities, and the proportion occurring at visits for different services, using a chi-square test. We compared the proportions of would-not-recommend visits when the man was the primary recipient versus the proportion of would-not-recommend visits when he was a caregiver (accompanying another person primarily for the other person’s health) with a test of equality of proportions. Third, we investigated which specific quality problems were associated with an overall would-not-recommend experience at the most recent visit. We conducted univariable logistic regressions to observe associations between would-not-recommend visits and individual problems from the Demographic and Health Surveys’ Service Provision Assessment scale. We then conducted a multivariable regression to identify which problems associated at a *p* < 0.05 level in univariable analyses were independently associated with a would-not-recommend visit and thus qualified as “high-priority” quality domains.

To understand the consequences of negative experiences, we examined health-seeking behavior after would-not-recommend visits 12–24 months previously. Our fourth analysis used logistic regressions to show the proportions of men who had any visit in the last 12 months and who returned to specific facilities in the past 12 months after acceptable or would-not-recommend experiences 12–24 months prior. Finally, in our fifth analysis, we tested the association between having a would-not-recommend visit 12–24 months ago and HIV testing behavior in the last 12 months using univariable and multivariable regressions. We controlled for other variables associated with HIV testing, including sociodemographic characteristics and gender norm beliefs. The multivariable model was a logistic regression with random effects to account for clustering at the village level. Because men were only asked about their four most recent visits, and all of these visits occurred in the last 12 months for some men, there are four groups of participants in the last two analyses: men with only positive visits reported 12–24 months ago; men with would-not-recommend visits 12–24 months ago; men with no visits 12–24 months ago; and men with four visits in the last 12 months (and therefore no data about visits 12–24 months ago).

All analyses were completed in Stata v.17 [31]. 

The parent study was approved by the National Health Sciences Review Committee (NHSRC) of Malawi (#2338) and the University of California Los Angeles (UCLA) Institutional Review Board (#20–001606).

## Results

A total of 1,187 men were enrolled in the parent study, ages 15–24 years (*n* = 300); 25–39 years (*n* = 425); and *≥* 40 years (*n* = 425). For this secondary analysis, we excluded 57 (4.8%) who reported knowing they were living with HIV more than 12 months prior to completing the survey and 32 (2.7%) who had not visited a health facility in the past 24 months (and thus had not reported on service satisfaction), with a total of 1,098 men in our analysis.

## Exploring Client Experiences and Satisfaction

Of the 1,098 men who attended a health facility in the past 24 months, 102 (9%) had a would-not-recommend visit (Table 1). Men were a mean age of 34 years, predominantly married (75%), and lived a mean of 4.96 km from a health facility. One-third (32%) reported having some form of financial savings, with a higher proportion among those with at least one would-not-recommend visit (41% vs. 31%). Less than a quarter (21%) ever attended secondary school. Most (84%) reported good or very good health status at the time of the survey. There were no significant differences in demographic characteristics between men with or without a would-not-recommend visit.

**Table 1:** Socio-demographic characteristics, gender norm beliefs, and HIV stigma beliefs of HIV-negative Malawian men with and without “would-not-recommend” experiences at health facilities in the last 24 months (*n* = 1098)

	Total men with any visit in last 24 months *n* = 1098 (100%)	Men with only acceptable visits *n* = 996 (91%)	Men with at least one would-not-recommend visit *n* = 102 (9%)	z (Wilcoxon rank sum)	t (t-test)	*p*-value
*Socio-demographic characteristics*						
Age (years, median)	34	34	34	0.93		0.35
Married (monogamous or polygamous) (n, %)	821 (75%)	742 (74%)	79 (77%)		-0.65	0.08
Distance from health facility (km, mean)	4.96	4.93	5.22	-0.94		0.35
*Economic indicators*						
Assets (index created by PCA; mean score)	1.89	1.89	1.89	0.07		0.95
Has savings (n, %)	355 (32%)	313 (31%)	42 (41%)		-2.01	0.08
Attended secondary school (n, %)	226 (21%)	205 (21%)	21 (21%)		-0.00	0.97
*Health / HIV risk factors*						
Sexual partners in last 12 months (mean)	1.46	1.45	1.54	-1.11		0.27
Good/very good health (vs. poor/very poor) (n, %)	924 (84%)	843 (85%)	81 (79%)		1.38	0.17
*Harmful gender norm beliefs*						
Violence scale, high	266 (24%)	247 (25%)	19 (19%)		1.39	0.17
Dominance scale, high	225 (20%)	208 (21%)	17 (17%)		1.00	0.32
Women’s roles scale, high	306 (28%)	285 (29%)	21 (21%)		1.72	0.09
Decision-making scale, high	308 (28%)	285 (29%)	23 (23%)		1.30	0.19
*HIV stigma beliefs*						
High HIV-related stigma	240 (22%)	217 (22%)	23 (23%)		-0.18	0.86

In total, men made 2,999 visits to health facilities; 2,852 (95%) were regarded as acceptable, i.e. men would recommend the facility to friends or family (Table 2). Proportions of would-not-recommend visits were different across facility type and service received. 20% of visits to private facilities were rated would-not-recommend versus 4% at government and mission facilities. Among OPD and dentist visits, 6% and 9% were rated would-not-recommend respectively, while 1% of visits for HIV services and for maternal and child health were rated would-not-recommend. Men were the primary service recipient at 1,832 visits and caregivers at 1,167 visits, and there was no difference in acceptable vs. would-not-recommend visits by service recipient.

**Table 2:** Distribution of acceptable and “would-not-recommend” visits across facility type, visit type, and care recipient as reported by HIV-negative Malawian men (*n* = 2999)

	All visits *n* = 2999	Acceptable visits *n* = 2852 (95%)	Would-not-recommend visits *n* = 147 (5%)	Chi-2 or z (*p*-value)
*Facility type*				Chi-2 = 70.03 (< 0.001)
Public (government)	2366	2263 (96%)	103 (4%)	
Mission or NGO	498	480 (96%)	18 (4%)	
Private	132	106 (80%)	26 (20%)	
Doesn’t remember	2	2 (67%)	1 (33%)	
*Service received*				Chi-2 = 18.49 (< 0.001)
OPD (injury or illness)	2465	2328 (94%)	137 (6%)	
HIV-related ^a^	314	310 (99%)	4 (1%)	
Maternal/child health ^b^	143	142 (99%)	1 (1%)	
Dentist	34	31 (91%)	3 (9%)	
Family planning	6	6 (100%)	0 (0%)	
Other or missing	37	36 (97%)	1 (3%)	
*Primary service recipient*				z = 1.53 (0.13)
Self	1832	1751 (96%)	91 (4%)	
Other (caregiver)	1167	1101 (94%)	66 (6%)	

^a^Includes HIV testing and counseling (HTC) and antiretroviral therapy (ART) clinic

^b^Includes antenatal, delivery, post-natal, and under-5 visits

*Note* percentages are given by row to reflect proportion of visits reported as would-not-recommend.

The most common problems with men’s most recent facility visit were wait time (21% of visits) and medicine availability (14% of visits) (Table 3). Four components of quality were strongly associated with reporting would-not-recommend in adjusted models: cost, cleanliness, wait time, and medicine availability. Men who reported major problems with cost (aOR 5.56, 95%CI 2.96–10.44) or cleanliness (aOR 4.17, 95%CI 1.87–9.33) were four to five times more likely to have a would-not-recommend visit than those who did not experience these major problems. Those who reported major problems with wait times (aOR 2.62, 95%CI 1.44–4.79) or medicine availability (aOR 2.72, 95% CI 1.47–5.04) were nearly three times more likely to have a would-not-recommend visit. Four other quality components (provider treatment, days of service, hours of service, and privacy of exam) were associated with would-not-recommend visits in univariable but not multivariable analysis. Problems varied by facility type: cost was reported as a problem more often at visits to mission and private facilities, while medicine availability was a more frequent problem at public facilities (Supplement 1).

**Table 3:** Crude and adjusted associations between specific facility problems and “would-not-recommend” experiences (*n* = 1084)

	Proportion of all visits (*n* = 1018)	Prop. of acceptable visits (*n* = 957)	Prop. of would-not-recommend visits (*n* = 61)	OR (95% CI)	aOR (95% CI)
*Major problem with*…					
Cost	88 (9%)	68 (7%)	20 (33%)	6.37 (3.54–11.49) **	5.56 (2.96–10.44) **
Cleanliness	46 (5%)	33 (3%)	13 (21%)	7.58 (3.75–15.34) **	4.17 (1.87–9.33) **
Wait time	217 (21%)	186 (19%)	31 (51%)	4.28 (2.53–7.25) **	2.62 (1.44–4.79) **
Medicine availability	145 (14%)	121 (13%)	24 (39%)	4.48 (2.59–7.75) **	2.72 (1.47–5.04) **
Provider treatment	57 (6%)	46 (5%)	11 (18%)	4.36 (2.13–8.92) **	1.36 (0.57–3.29)
Days of service	54 (5%)	45 (5%)	9 (15%)	3.51 (1.62–7.56) **	1.15 (0.43–3.09)
Hours of service	98 (10%)	84 (9%)	14 (23%)	3.09 (1.64–5.86) **	1.14 (0.51–2.58)
Privacy of exam	16 (2%)	13 (1%)	3 (5%)	3.75 (1.04–13.55) **	0.76 (0.14–4.15)
Privacy of discussion	15 (1%)	13 (1%)	2 (3%)	2.46 (0.54–11.16)	
Ability to discuss concerns	46 (5%)	41 (4%)	5 (8%)	1.99 (0.76–5.25)	

* p<0.10;

** p<0.05;

*** p<0.01

### Consequences of Would-Not-Recommend Experiences

There was no significant difference in returning to any facility for a visit in the last 12 months between men who reported a would-not-recommend visit 12–24 months ago and men who reported acceptable visits 12–24 months ago (OR 0.65; 95% CI 0.26–1.61; *p* = 0.27) (Table 4). However, men who reported a would-not-recommend visit 12–24 months ago were less likely to return to that same facility in the last 12 months (OR 0.43; 95% CI 0.19–0.95).

**Table 4:** Association between facility visits at any and same facility in last 12 months and experiences at facility visits 12–24 months ago among HIV-negative men in Malawi (*n* = 1098)

		*Any* facility visit in last 12 months	Returned to *same* facility in last 12 months
	*n* = 898	Odds ratio (95% CI)	Odds ratio (95% CI)
*Healthcare experiences at facilities 12-24mo ago*			
Acceptable visit	554 (50%)	1 (ref)	1 (ref)
Would-not-recommend visit	30 (3%)	0.65 (0.26–1.61)	0.43 (0.19–0.95)**
No visit 12–24 months ago	314 (29%) 200 (18%)	0.63 (0.44–0.91)** n/a	n/a n/a
Visit maximum reached (4 visits in last 12 months and no data on visits 12–24 months ago)	200 (18%)	n/a	n/a

* p<0.10;

** p<0.05;

*** p<0.01

For HIV testing services, men who had would-not-recommend visits 12–24 months ago were 59% less likely to test for HIV in the past 12 months than those with only acceptable experiences (aOR 0.41, 95% CI 0.17–0.96) when controlling for other factors associated with HIV testing (Table 5). Men with more than four visits in the last 12 months were three times more likely to have tested for HIV (aOR 3.24, 95% CI 2.23–4.69) than men with fewer visits who reported an acceptable visit 12–24 months ago.

**Table 5:** Crude and adjusted analysis of factors associated with receiving an HIV test in the last 12 months among men in need of testing in Malawi (*n* = 1098)

	Odds ratio	Adjusted odds ratio
*Healthcare experiences at facilities 12–24 months ago*		
Acceptable visit	1	1
Would-not-recommend visit	0.42 (0.19–0.97) **	0.41 (0.17–0.96) **
No visit 12–24 months ago	0.57 (0.43–0.76) ***	0.55 (0.41–0.75) ***
Visit maximum reached (4 visits in last 12 months and no data on visits 12–24 months ago)	3.40 (2.37–4.87) ***	3.24 (2.23–4.69) ***
*Socio-demographic characteristics*		
Age (years, median) ^a^	1.01 (1.00-1.02)	0.99 (0.98-1.00)
Married (monogamous or polygamous) (n, %)	1.93 (1.46–2.56) ***	1.88 (1.26–2.83) ***
*Economic indicators*		
Assets (index created by PCA; mean score)	1.03 (0.95–1.11)	
Has savings (n, %)	1.60 (1.24–2.07) ***	1.38 (1.04–1.82) **
Attended secondary school (n, %)	1.49 (1.11-2.00) ***	1.31 (0.94–1.82)
*Health / HIV risk factors*		
Number of sexual partners in last 12 months (mean)	1.16 (1.06–1.28) ***	1.13 (1.02–1.24) **
Good/very good health (vs. poor/very poor) (n, %)	0.80 (0.58–1.10)	
*Harmful gender norm beliefs*		
Violence scale, high	1.04 (0.79–1.37)	
Dominance scale, high	1.25 (0.94–1.69)	
Women’s roles scale, high	1.20 (0.92–1.56)	
Decision-making scale, high	1.29 (0.99–1.68)	
*HIV stigma beliefs*		
High HIV-related stigma	0.65 (0.49–0.87) ***	0.83 (0.60–1.17)

^a^ Included as a control regardless of significance in multivariable analysis

* p<0.10;

** p<0.05;

*** p<0.01

## Discussion

Our study confirms that men’s satisfaction with general health services matters and is key to future decisions about if and where to use health services. Poor client satisfaction was associated with problems due to cost, cleanliness, wait times, and medicine availability. Men who reported would-not-recommend visits had no significantly different characteristics from men who did not report would-not-recommend visits, suggesting that all men are at risk of negative experiences. Would-not-recommend experiences with general health services were negatively associated with men’s future HIV testing, even when controlling for other factors associated with HIV testing. Men were also less likely to return to the same health facility after a would-not-recommend visit, whether for HIV or non-HIV services, though they were not less likely to attend any facility overall.

Our study underlines the close link between HIV care and non-HIV services – men do not view healthcare in a silo. Negative experiences with general, non-HIV services can influence use of HIV-related services in the future. Although it has long been argued that men do not attend health care facilities, we have previously found that over 85% of Malawian men had attended an outpatient health visit at least once in the previous year for acute injury or illness [24]. A negative experience at an outpatient department may affect a man’s decision to use HIV testing because departments share infrastructure and staff, even if funders and monitoring and evaluation systems treat services as separate. If the client wishes to avoid negative interactions, he may feel safer avoiding that health facility or avoiding screening services until he feels sick. Thus it is critical that interventions to increase uptake of HIV testing account for the broader health system context. As we have shown, satisfaction with non-HIV services is associated with future HIV testing for Malawian men, highlighting the need for a holistic approach to health services rather than siloed programs that isolate one illness. Quality experiences within outpatient departments should be prioritized, as the majority of men’s interactions with health systems are within outpatient settings for acute injury or illness.

There are increasing calls to prioritize high quality, client-centered approaches for service delivery [32], and these should become a focus of health services research for men. With few exceptions [9, 17, 33, 34], most studies investigating men’s health-seeking behavior in sub-Saharan Africa do not account for perceived or real quality of care and client satisfaction [18, 19, 35], and only a small number of studies document women’s quality-based decisions for obstetric care [36, 37]. Our results confirm the critical importance of viewing clients – men and women – as active consumers rather than passive recipients of health services [10]. Future research on health-seeking behavior should account for the impact of previous negative experiences with both HIV and non-HIV services, and interventions designed to increase men’s care utilization should improve service quality or directly address previous negative experiences in male-specific counseling, such as a recent trial in Malawi has done [38]. Moreover, future research should rigorously evaluate whether gender norms play a measurable role in men’s healthcare utilization. Though various qualitative studies assert that gender norms explain men’s low utilization rates [18, 39], if this were true, we would expect to see lower utilization among men with the strongest gender norm beliefs, which was not the case in our data. Our survey data is not adequate to assess whether gender norm beliefs play *any* role in men’s healthcare experiences, but findings from another study using this dataset suggest that the effects of such norms are not significant when the analysis controls for the impact of service quality [9].

We identified several domains of quality services that were associated with poor client satisfaction, and we found that these problems were fairly common. Of all facility visits reported, 21% had a major problem with wait time and 14% had a major problem with medicine availability (i.e., medicine stockouts). Quality problems affected all types of facilities, as well. We did not expect that private facilities would have higher rates of would-not-recommend visits, but problems with cost – without compensatory quality improvements in, for example, provider treatment or wait times – were powerful drivers of dissatisfaction in this population. Improving quality across these common problem areas may increase demand for high-priority screening services like HIV testing.

The temporal aspect of our study is a key strength: we identified past healthcare experiences and their association with subsequent service uptake. However, our data may suffer from recall bias, which may partly explain the small number of negative experiences reported. As this is a secondary analysis, the study was not powered to find differences between men who did and did not report would-not-recommend visits, so our null result in the first analysis may reflect inadequate sample size rather than true equivalence between the two groups. This small sample also affected our ability to determine the specific mechanism leading to an association between dissatisfaction and lower HIV testing. Future research should explore several hypotheses to explain how dissatisfaction leads to lower testing: men may specifically avoid preventative services after previous negative experiences; providers may be less likely to offer tests to mistrustful clients; or men may be less likely to accept tests after previous negative experiences. Qualitative research on men’s healthcare experiences will be especially important to explore these topics, but we believe that our study has shown that the issues can be assessed quantitatively as well.

Men’s suboptimal uptake of health services, especially screening services like HIV testing, is influenced by men’s previous satisfaction with general healthcare services. Overall health system quality may drive men’s health-seeking behavior and should be improved to increase uptake of priority services.

### Electronic supplementary material

Below is the link to the electronic supplementary material.

Supplementary Material 1

## Data Availability

The data and code that support the findings of this study are available from the corresponding author, MT, upon reasonable request. Due to the sensitive nature of data regarding HIV status, data will not be deposited in a public repository.

## Appendix A. Map of health facilities selected for community-representative survey

**Appendix B.** Gender norm measures and questions

Measure	Question
Violence is permissible (violence scale)	There are times when a woman deserves to be beaten. A woman should tolerate violence in order to keep her family together. If someone insults me, I will defend my reputation, with force if I have to.
Male sexual dominance is acceptable (dominance scale)	Men need sex more than women. Men don’t talk about sex, they just do it. Men are always ready to have sex.
Women’s roles should be confined to the household (women’s roles scale)	Changing diapers, giving kids a bath & feeding kids are mother’s responsibility, It is a woman’s responsibility to avoid getting pregnant. A man should have the final word about decisions in his home.
Men control decisions in participant’s household (decision-making scale)	Who usually decides how the money you earn will be used? Who usually decides how your partner's earnings will be used? Who usually makes decisions about making major household purchases?

## References

[1] Global Health Observatory. In: Organization WH, editor. Geneva2018.

[2] Baker P, Dworkin SL, Tong S, Banks I, Shand T, Yamey G. The men’s health gap: men must be included in the global health equity agenda. Bull World Health Organ. 2014;92(8):618–20. 10.2471/blt.13.132795. Epub 2014/09/10. 25197149 10.2471/blt.13.132795 PMC4147416

[3] Hawkes S, Buse K. Gender and global health: evidence, policy, and inconvenient truths. Lancet. 2013;381(9879):1783–7. 10.1016/s0140-6736(13)60253-6. Epub 2013/05/21. 23683645 10.1016/s0140-6736(13)60253-6

[4] UNAIDS. Blind spot: reaching out to men and boys. Addressing a blind spot in the response to HIV. Switzerland: UNAIDS Geneva; 2017.

[5] Osler M, Cornell M, Ford N, Hilderbrand K, Goemaere E, Boulle A. Population-wide differentials in HIV service access and outcomes in the Western Cape for men as compared to women, South Africa: 2008 to 2018: a cohort analysis. J Int AIDS Soc. 2020;23(Suppl 2):e25530. 10.1002/jia2.25530. Epub 2020/06/27. 32589367 10.1002/jia2.25530 PMC7319137

[6] Dovel K, Yeatman S, Watkins S, Poulin M. Men’s heightened risk of AIDS-related death: the legacy of gendered HIV testing and treatment strategies. AIDS. 2015;29(10):1123–5. 10.1097/qad.0000000000000655. PubMed PMID: 00002030-201506190-00003. 26035315 10.1097/qad.0000000000000655 PMC4454403

[7] Druyts E, Dybul M, Kanters S, Nachega J, Birungi J, Ford N, Thorlund K, Negin J, Lester R, Yaya S, Mills EJ. Male sex and the risk of mortality among individuals enrolled in antiretroviral therapy programs in Africa: a systematic review and meta-analysis. AIDS. 2013;27(3):417–25. 10.1097/QAD.0b013e328359b89b. PubMed PMID: 00002030-201301280-00012. 22948271 10.1097/QAD.0b013e328359b89b

[8] Monod M, Brizzi A, Galiwango RM, Ssekubugu R, Chen Y, Xi X, Kankaka EN, Ssempijja V, Dörner LA, Akullian A, Blenkinsop A, Bonsall D, Chang LW, Dan S, Fraser C, Golubchik T, Gray RH, Hall M, Jackson JC, Kigozi G, Laeyendecker O, Mills LA, Quinn TC, Reynolds SJ, Santelli J, Sewankambo NK, Spencer SE, Ssekasanvu J, Thomson L, Wawer MJ, Serwadda D, Godfrey-Faussett P, Kagaayi J, Grabowski MK, Ratmann O. Growing gender disparity in HIV infection in Africa: sources and policy implications. medRxiv. 2023. 10.1101/2023.03.16.23287351. Epub 2023/03/31. 36993261 10.1101/2023.03.16.23287351 PMC10055554

[9] Thorp M, Balakasi K, Robson MM, Khan I, Stillson S, Doi C, Nichols N, Dovel BE. Factors associated with men’s health facility attendance as clients and caregivers in Malawi: a community-representative survey. BMC Public Health. 2022;22:1904. 36224573 10.1186/s12889-022-14300-8 PMC9558411

[10] Leonard KL. Active patients in rural African health care: implications for research and policy. Health Policy Plan. 2014;29(1):85–95. 10.1093/heapol/czs137. Epub 2013/01/12. 23307907 10.1093/heapol/czs137

[11] Kwame A, Petrucka PM. Communication in nurse-patient interaction in healthcare settings in sub-saharan Africa: a scoping review. Int J Afr Nurs Sci. 2020;12:100198. 10.1016/j.ijans.2020.100198. 10.1016/j.ijans.2020.100198

[12] Camara BS, Belaid L, Manet H, Kolie D, Guillard E, Bigirimana T, Delamou A. What do we know about patient-provider interactions in sub-saharan Africa? A scoping review. Pan Afr Med J. 2020;37:88. 10.11604/pamj.2020.37.88.24009. Epub 2020/11/28. 33244351 10.11604/pamj.2020.37.88.24009 PMC7680249

[13] Ratcliffe HL, Bell G, Awoonor-Williams K, Bitton A, Kim JH, Lipstiz S, Macarayan E, Ofosu A, Otupiri E, Schwarz D, Hirschhorn LR. Towards patient-centred care in Ghana: health system responsiveness, self-rated health and experiential quality in a nationally representative survey. BMJ Open Qual. 2020;9(2). Epub 2020/05/15. 10.1136/bmjoq-2019-000886. PubMed PMID: 32404309; PMCID: PMC7228562. 10.1136/bmjoq-2019-000886 PMC7228562 32404309

[14] Anabila P, Kumi DK, Anome J. Patients’ perceptions of healthcare quality in Ghana. Int J Health Care Qual Assur. 2019;32(1):176–90. 10.1108/ijhcqa-10-2017-0200. Epub 2019/03/13. 30859879 10.1108/ijhcqa-10-2017-0200

[15] Gage AD, Leslie HH, Bitton A, Jerome JG, Joseph JP, Thermidor R, Kruk ME. Does quality influence utilization of primary health care? Evidence from Haiti. Global Health. 2018;14(1):59. 10.1186/s12992-018-0379-0. Epub 2018/06/22. 29925416 10.1186/s12992-018-0379-0 PMC6011404

[16] Kruk ME, Rockers PC, Mbaruku G, Paczkowski MM, Galea S. Community and health system factors associated with facility delivery in rural Tanzania: a multilevel analysis. Health Policy. 2010;97(2–3):209 – 16. Epub 2010/06/12. doi: 10.1016/j.healthpol.2010.05.002. PubMed PMID: 20537423. 10.1016/j.healthpol.2010.05.002 20537423

[17] Nangendo J, Katahoire AR, Armstrong-Hough M, Kabami J, Obeng-Amoako GO, Muwema M, Semitala FC, Karamagi CA, Wanyenze RK, Kamya MR, Kalyango JN. Prevalence, associated factors and perspectives of HIV testing among men in Uganda. PLoS ONE. 2020;15(8):e0237402. 10.1371/journal.pone.0237402. 32764820 10.1371/journal.pone.0237402 PMC7413494

[18] Sileo KM, Fielding-Miller R, Dworkin SL, Fleming PJ. What role do masculine norms play in men’s HIV Testing in Sub-saharan Africa? A scoping review. AIDS Behav. 2018;22(8):2468–79. 10.1007/s10461-018-2160-z. 29777420 10.1007/s10461-018-2160-z PMC6459015

[19] Fleming PJ, Rosen JG, Wong VJ, Carrasco MA. Shedding light on a HIV blind spot: factors associated with men’s HIV testing in five African countries. Glob Public Health. 2019;14(9):1241–51. 10.1080/17441692.2019.1583266. 30794471 10.1080/17441692.2019.1583266

[20] Camlin CS, Ssemmondo E, Chamie G, El Ayadi AM, Kwarisiima D, Sang N, Kabami J, Charlebois E, Petersen M, Clark TD, Bukusi EA, Cohen CR, Havlir MRK. Men missing from population-based HIV testing: insights from qualitative research. AIDS Care. 2016;28(Suppl 3):67–73. PubMed PMID: 27421053; PMCID: PMC5749410. 27421053 10.1080/09540121.2016.1164806 PMC5749410

[21] Tibbels NJ, Hendrickson ZM, Naugle DA, Dosso A, Van Lith L, Mallalieu EC, Kouadio AM, Kra W, Kamara D, Dailly-Ajavon P, Cisse A, Seifert-Ahanda K, Thaddeus S, Babalola S, Hoffmann CJ. Men’s perceptions of HIV care engagement at the facility- and provider-levels: experiences in Cote d’Ivoire. PLoS ONE. 2019;14(3):e0211385. 10.1371/journal.pone.0211385. 30897098 10.1371/journal.pone.0211385 PMC6428322

[22] Layer EH, Kennedy CE, Beckham SW, Mbwambo JK, Likindikoki S, Davis WW, Kerrigan DL, Brahmbhatt H, the LTCTCST. Multi-level factors affecting entry into and Engagement in the HIV Continuum of Care in Iringa, Tanzania. PLoS ONE. 2014;9(8):e104961. 10.1371/journal.pone.0104961. 25119665 10.1371/journal.pone.0104961 PMC4138017

[23] Osborn M, Obermeyer CM. Understanding client satisfaction with HIV testing and counseling services: a mixed-methods study in four African countries. AIDS Care. 2016;28(6):689–94. 10.1080/09540121.2016.1140889. Epub 2016/02/14. 26872848 10.1080/09540121.2016.1140889

[24] Dovel K, Balakasi K, Gupta S, Mphande M, Robson I, Khan S, Amberbir A, Stilson C, van Oosterhout JJ, Doi N, Nichols BE. Frequency of visits to health facilities and HIV services offered to men. Malawi Bull World Health Organ. 2021;99(9). 10.2471/BLT.20.278994 PMC8381098 34475599

[25] Nhassengo P, Cataldo F, Magaço A, Hoffman RM, Nerua L, Saide M, Cuco R, Hoek R, Mbofana F, Couto A, Gudo E, Chicumbe S, Dovel K. Barriers and facilitators to the uptake of test and treat in Mozambique: a qualitative study on patient and provider perceptions. PLoS ONE. 2018;13(12):e0205919. 10.1371/journal.pone.0205919. 30586354 10.1371/journal.pone.0205919 PMC6306230

[26] Filmer D, Pritchett LH. Estimating wealth effects without expenditure data–or tears: an application to educational enrollments in states of India. Demography. 2001;38(1):115–32. 10.1353/dem.2001.0003. Epub 2001/03/03. 11227840 10.1353/dem.2001.0003

[27] Shattuck D, Burke H, Ramirez C, Succop S, Costenbader B, Attafuah JD, Mndeme E, Mbwambo J, Guest G. Using the inequitable gender norms Scale and Associated HIV Risk behaviors among men at high risk for HIV in Ghana and Tanzania. Men Masculinities. 2013;16(5):540–59. 10.1177/1097184x13502730. 10.1177/1097184x13502730

[28] Phiri K, McBride K, Moucheraud C, Mphande M, Balakasi K, Lungu E, Kalande P, Hoffman RM, Dovel K. Community and health system factors associated with antiretroviral therapy initiation among men and women in Malawi: a mixed methods study exploring gender-specific barriers to care. Int Health. 2021;13(3):253–61. 10.1093/inthealth/ihaa041. Epub 2020/08/28. 32844205 10.1093/inthealth/ihaa041 PMC8079311

[29] Lewis S, Moucheraud C, Schechinger D, Mphande M, Banda BA, Sigauke H, Kawale P, Dovel K, Hoffman RM. A loving man has a very huge responsibility: a mixed methods study of Malawian men’s knowledge and beliefs about cervical cancer. BMC Public Health. 2020;20(1):1494. 10.1186/s12889-020-09552-1. Epub 2020/10/04. 33008344 10.1186/s12889-020-09552-1 PMC7532091

[30] Program DaHS. Service Provision Assessment: Sick Child Caretaker Exit Interview (SPAQ3) 2012 [7 December 2022]. https://dhsprogram.com/pubs/pdf/SPAQ3/SICK_CHILD_CARETAKER_EXIT_INTERVIEW_09112020.pdf.

[31] StataCorp. Stata Statistical Software: release 14. College Station. TX: StataCorp LP; 2015.

[32] Lazarus JV, Safreed-Harmon K, Kamarulzaman A, Anderson J, Leite RB, Behrens G, Bekker L-G, Bhagani S, Brown D, Brown G, Buchbinder S, Caceres C, Cahn PE, Carrieri P, Caswell G, Cooke GS, Monforte AA, Dedes N, del Amo J, Elliott R, El-Sadr WM, de Fuster-Ruiz MJ, Guaraldi G, Hallett T, Harding R, Hellard M, Jaffar S, Kall M, Klein M, Lewin SR, Mayer K, Pérez-Molina JA, Moraa D, Naniche D, Nash D, Noori T, Pozniak A, Rajasuriar R, Reiss P, Rizk N, Rockstroh J, Romero D, Sabin C, Serwadda D, Waters L. Consensus statement on the role of health systems in advancing the long-term well-being of people living with HIV. Nat Commun. 2021;12(1):4450. 10.1038/s41467-021-24673-w. 34272399 10.1038/s41467-021-24673-w PMC8285468

[33] Hlongwa M, Mashamba-Thompson T, Makhunga S, Hlongwana K. Barriers to HIV testing uptake among men in sub-saharan Africa: a scoping review. Afr J AIDS Res. 2020;19(1):13–23. 10.2989/16085906.2020.1725071. 32174231 10.2989/16085906.2020.1725071

[34] Mak J, Mayhew SH, von Maercker A, Colombini IRT. Men’s use of sexual health and HIV services in Swaziland: a mixed methods study. Sex Health. 2016;13(3):265–74. 10.1071/SH15244. 27028455 10.1071/SH15244

[35] Stephenson R, Miriam Elfstrom K, Winter A. Community influences on Married men’s Uptake of HIV Testing in eight African countries. AIDS Behav. 2013;17(7):2352–66. 10.1007/s10461-012-0223-0. 22677974 10.1007/s10461-012-0223-0 PMC3903034

[36] Tiruneh GT, Demissie M, Worku A, Berhane Y. Community’s experience and perceptions of maternal health services across the continuum of care in Ethiopia: a qualitative study. PLoS ONE. 2021;16(8):e0255404. 10.1371/journal.pone.0255404. Epub 2021/08/05. 34347800 10.1371/journal.pone.0255404 PMC8336848

[37] Moyer CA, Mustafa A. Drivers and deterrents of facility delivery in sub-saharan Africa: a systematic review. Reprod Health. 2013;10:40. 10.1186/1742-4755-10-40. Epub 2013/08/22. 23962135 10.1186/1742-4755-10-40 PMC3751820

[38] Robson I, Hubbard J, Mphande M, Thorp M, Phiri K, Daniels J, Lee J, Chikuse E, Cornell M, Dovel K. Implementing a male-specific ART counseling curriculum: a quality assessment with HCWs in Malawi. Under review, JIAS2023. 10.1002/jia2.26270 PMC11263468 39039724

[39] Chikovore J, Hart G, Kumwenda M, Chipungu GA, Corbett L. ‘For a mere cough, men must just chew Conjex, gain strength, and continue working’: the provider construction and tuberculosis care-seeking implications in Blantyre, Malawi. Glob Health Action. 2015;8:26292. Epub 2015/04/04. 10.3402/gha.v8.26292. PubMed PMID: 25833138; PMCID: PMC4382597. 10.3402/gha.v8.26292 PMC4382597 25833138

