# Supplementary figures and images for: Men’s Satisfaction with General Health Services is Associated with Future Use of HIV Testing in Malawi: A Community-Representative Survey
Source: AIDS Behav. 2024 Jun 13;28(8):2639–49. doi: 10.1007/s10461-024-04352-9 (PMC11286645; doi:10.1007/s10461-024-04352-9)

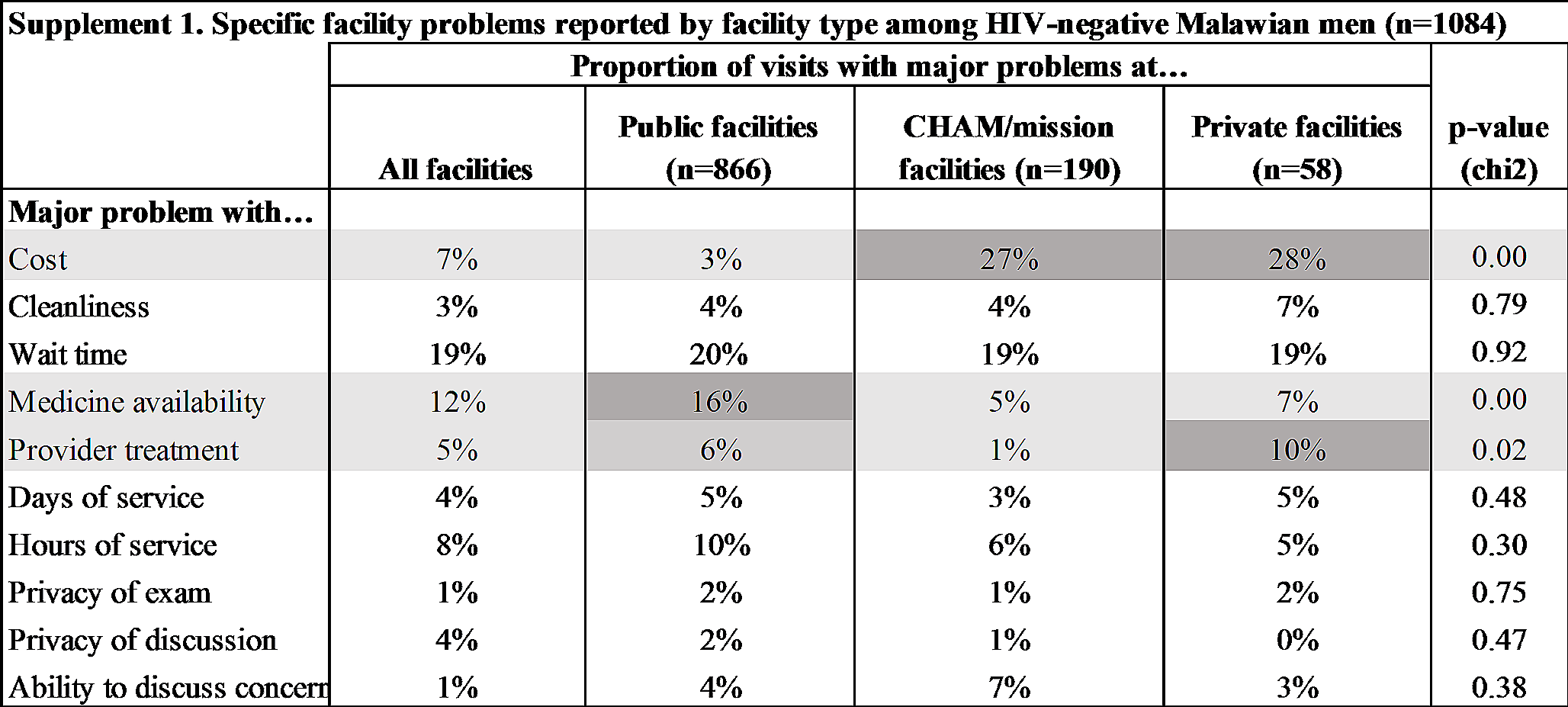

Supplement: Supplementary file 1 — Supplementary Material 1 [file 10461_2024_4352_MOESM1_ESM.docx]
